# Visualizing the Immune System: Providing Key Insights into HIV/SIV Infections

**DOI:** 10.3389/fimmu.2018.00423

**Published:** 2018-03-02

**Authors:** Jacob D. Estes, Roger LeGrand, Constantinos Petrovas

**Affiliations:** ^1^Vaccine and Gene Therapy Institute, Oregon Health & Science University, Beaverton, OR, United States; ^2^Oregon National Primate Research Center, Oregon Health & Science University, Beaverton, OR, United States; ^3^CEA, Université Paris Sud 11, INSERM U1184, Center for Immunology of Viral Infections and Autoimmune Diseases, IDMIT Department, IBFJ, Fontenay-aux-Roses, France; ^4^Tissue Analysis Core, Vaccine Research Center, National Institute of Allergy and Infectious Diseases (NIAID) National Institutes of Health (NIH), Bethesda, MD, United States

**Keywords:** HIV, lymph nodes, mucosa, immune cells, T cells, imaging

## Abstract

Immunological inductive tissues, such as secondary lymphoid organs, are composed of distinct anatomical microenvironments for the generation of immune responses to pathogens and immunogens. These microenvironments are characterized by the compartmentalization of highly specialized immune and stromal cell populations, as well as the presence of a complex network of soluble factors and chemokines that direct the intra-tissue trafficking of naïve and effector cell populations. Imaging platforms have provided critical contextual information regarding the molecular and cellular interactions that orchestrate the spatial microanatomy of relevant cells and the development of immune responses against pathogens. Particularly in HIV/SIV disease, imaging technologies are of great importance in the investigation of the local interplay between the virus and host cells, with respect to understanding viral dynamics and persistence, immune responses (i.e., adaptive and innate inflammatory responses), tissue structure and pathologies, and changes to the surrounding milieu and function of immune cells. Merging imaging platforms with other cutting-edge technologies could lead to novel findings regarding the phenotype, function, and molecular signatures of particular immune cell targets, further promoting the development of new antiviral treatments and vaccination strategies.

## Introduction

Investigation of the human immune system in the context of infectious diseases has been accomplished primarily based on studies utilizing circulating cells. However, use of such biological material may not capture the *in vivo* timing or mechanisms governing the initiation and development of immune responses to pathogens at important anatomical sites, such as secondary lymphoid organs, mucosal-associated lymphoid tissues (MALTs), and mucosae. Therefore, the need for comprehensive analysis of tissues central to disease pathogenesis, and interactions between theses tissues, is of great importance. The application of multidimensional methodologies, like polyparametric flow cytometry, has provided critical information regarding the phenotype and functionality of tissue-resident immune cells, especially T and B cells (1–5). Despite their analytical power, these methodologies cannot address the tissue distribution/localization of lymphoid populations *in vivo*, as well as the anatomical context in which their highly dynamic interactions occur. On the other hand, tissue investigation using histopathological assays, like immunohistochemistry, has provided critical information regarding the impact of HIV/SIV on the organization of the human immune system at a tissue level (6–14).

Imaging technologies are continuingly advancing, with new hardware (i.e., new types of cameras, laser lines, hybrid detectors, etc.) and software, improving the quality of images obtained at the level of acquisition, segmentation, and deconvolution of cells. Furthermore, the availability of steadily increasing antibody specificities and appropriate labels/probes further facilitates the application of imaging technologies to biological material. The introduction of advanced imaging technologies, such as multispectral confocal (15) and multiphoton microscopy, as well as imaging mass cytometry, positron emission tomography (PET), and magnetic resonance imaging (MRI) (16–18), opens new opportunities for the investigation of molecular and cellular events at dimensions that range from the nanoscale to the entire body and for visualizing the dynamic changes occurring in living tissues and individuals. Furthermore, the availability of technologies like stimulated emission depletion microscopy (STED) can provide unprecedented resolution (~20–50 nm) using light microscopy (19) for the detailed analysis and quantification of molecular dynamics at a subcellular level (20). Therefore, the application of cutting-edge imaging technologies can provide substantial novel insights into host–pathogen interactions that are simply not feasible with other approaches (Table 1), which may be critical for the development of vaccines, especially those aiming to elicit broadly neutralizing antibodies, as well as for the discovery of novel immunotherapy targets to eliminate HIV.

**Table 1:** Importance of performing tissue imaging.

– Cells in their natural environment – Tissue architecture, stromal cells – Compartmentalization of immune reactivity (immune cells, soluble factors) – Complexity of local immune dynamics – Displacement of cells and duration of local interactions – Dynamics and mechanisms of virus transmission – Generation of new questions – Tissue pathology and damage

## Why Do We Need Tissue Imaging?

Secondary lymphoid organs (i.e., lymph nodes and spleen) and MALT create an extended tissue network that provides a unique microenvironment for pathogen capture, antigen presentation, and induction of adaptive immune responses (21–23). The *ex vivo* and *in vitro* analysis of cells derived from such tissues using powerful methodologies like polyparametric flow cytometry and sequencing of sorted cell subsets has provided important information about the character and molecular profile of cells involved in the development of these responses (4, 15, 24, 25). The application of imaging technologies, however, can provide relevant information about cell populations in their “natural environment” and with respect to their spatial positioning, displacement, surrounding cells, and milieu microenvironment. Furthermore, estimating the possible role of parameters, like cell shape and polarization (26), in the biological process under investigation is impossible for cells removed from their natural tissue microenvironment. To this end, the combination of *ex vivo* organ culture models (27) with imaging analysis and whole-body *in vivo* studies would significantly increase our knowledge about the role of particular cells and soluble factors in HIV/SIV pathogenesis.

The compromised immune response against pathogens in subjects with genetic defects that affect the architecture and development of follicles demonstrates the importance of tissue integrity for an effective response against pathogens (28–30). It is well established that HIV/SIV infections are associated with extensive changes/damage of tissue architecture, especially in LNs and gut mucosa (31). Stromal cells, like fibroblastic reticular cells (FRCs) and follicular dendritic cells (FDCs), represent critical elements of the lymphoid tissue architecture, which are significantly affected by HIV/SIV (32–35), and because of their biology and function forming extended interdigitating networks within the follicular (FDC) (36) and extra-follicular (FRC) (37) areas, their isolation and *in vitro* analysis is challenging. Thus, imaging these stromal elements in their native intact tissue environments, with 3D volumetric analysis, will likely be essential to fully understand the importance of these networks in HIV/SIV infections. A comprehensive understanding of tissue perturbations in terms of cellularity and architecture will further elucidate defects in adaptive cellular responses and in the generation of antibody responses with functionalities that effectively control the virus, including broadly neutralizing antibodies.

**Figure 1:**
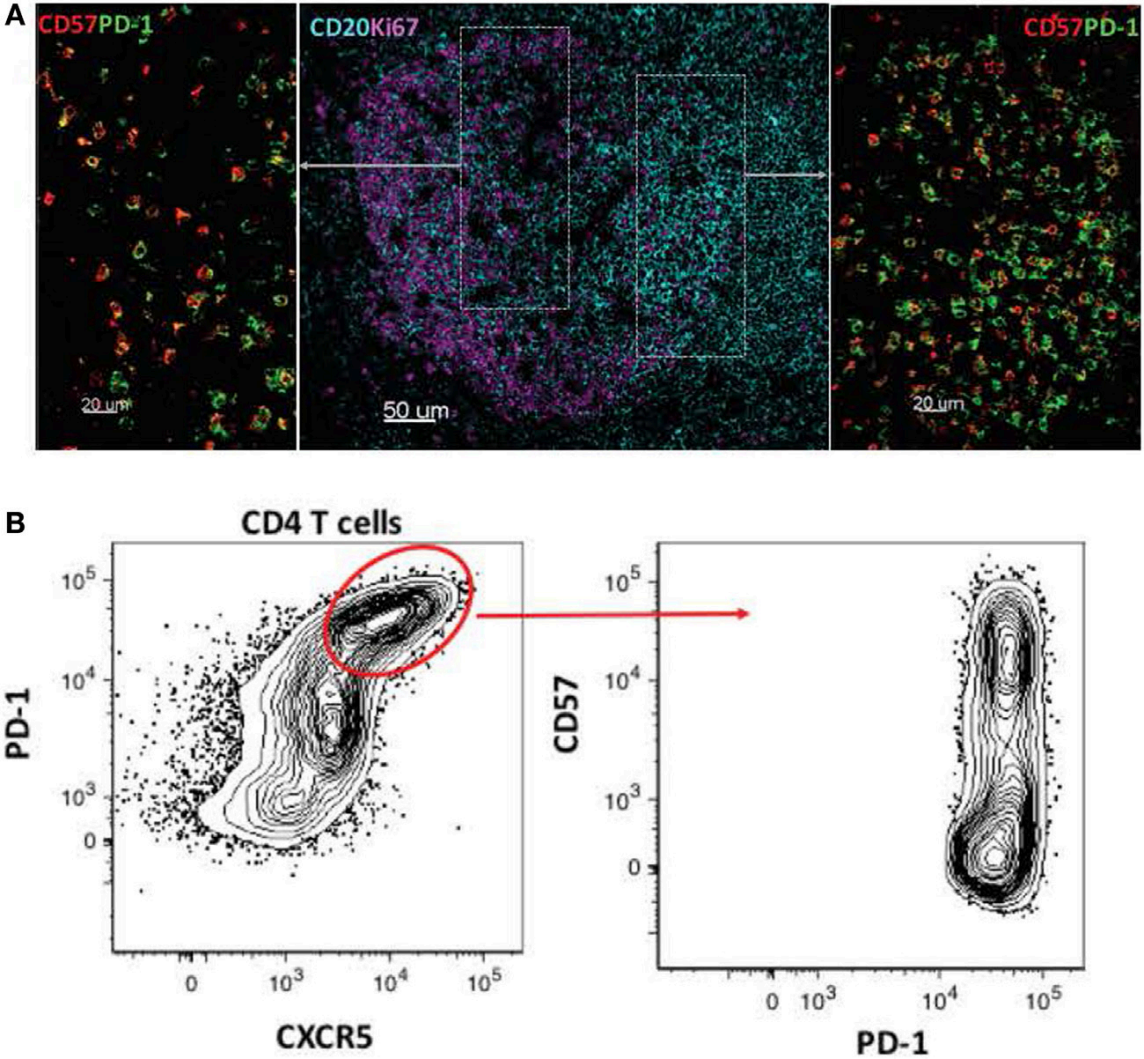
Heterogeneity of follicular cell populations. **(A)** Confocal images showing the relative distribution of proliferating B cells (CD20hi/dimKi67hi, CD20/cyan, and Ki67/magenta) and CD4 Tfh subsets (PD-lhiCD57hi, PD-lhiCD57lo, PD-1/green, and CD57/red) in a tonsillar follicular area. **(B)** Flow-cytometry plots showing the phenotype of tonsillar Tfh subsets based on the combined expression of PD-1, CXCR5, and CD57 surface receptors.

The development of effective adaptive immune responses against pathogens is a multistep process that requires the orchestrated function of several cell types and soluble factors within the LN environment. A critical aspect of this process is the compartmentalization of immune cell subsets with different origins or maturation status, as well as the presence of chemokine gradients that direct this compartmentalization and trafficking of cells between and within areas of LN. For example, the development of high-affinity, antigen-specific B-cell responses requires interactions between CD4 + T cells and B cells in the follicle. The identification of human follicular helper CD4 + T cells (Tfh) revealed a highly specialized CD4 + T-cell subset with a unique phenotypic, functional, and molecular signature (38–40). Still, Tfh cells represent a heterogeneous cellular population with different combinations of expressed surface receptors, such as PD-1, CD150, and CD57 (4, 41, 42). Likewise, follicular B cells represent a diverse population with different phenotypic profiles depending on their localization in germinal cell areas [light and dark zone (43, 44)]. Analysis of these populations based on their phenotype using flow-cytometry assays has been particularly informative with respect to their relative frequencies and dynamics in human and animal disease models. However, their phenotype does not always indicate their localization within tissue microenvironments. For example, although the dark zone is the site where B-cell division takes place, many proliferating (Ki67 +) B cells can be found in the light zone, and under physiological conditions, Tfh subsets have a distinct localization pattern (Figure 1). This type of imaging analysis can provide additional unique information regarding the juxtaposition/clustering of Tfh cells and B cells, the “polarization” pattern of germinal centers, as well as the distribution of Tfh cells, B cells, and FDCs within these LN follicular areas. Investigation of the impact that HIV/SIV infection has on the microanatomy of tissue environments could provide information about the cellular and molecular mechanisms mediating the development of humoral responses, as well as the local interplay between the host and virus during HIV/SIV disease progress.

The introduction of novel imaging technologies could generate new perspectives regarding the role of particular immune cell subsets. Traditionally, the quality of CD8 + T cells in HIV/SIV infection has been evaluated based on (i) their capacity to produce multiple cytokines (poly-functionality) (45), (ii) the expression of an “exhausted” phenotype related to their function and survival/proliferative capacity (46–48), and (iii) their potential for killing infected targets, mainly through perforin/granzyme protein expression (49, 50). However, an effective CTL response requires trafficking of activated and differentiated CD8 + T cells in areas with HIV/SIV-infected cells, followed by their ability to sense, efficiently engage the infected cells and perform their CTL effector function. Development of imaging-based methods allowing for the evaluation of such biological processes/steps (10) could provide a comprehensive analysis of CTL responses in HIV/SIV infection and contribute to a holistic view of the efficiency of adaptive responses needed for virus elimination.

## What Can We Learn from HIV/SIV Infections?

### The Host: Lymph Nodes

Classic immunohistochemistry studies have provided valuable information regarding the impact of HIV/SIV infection on the structure of tissues, such as LNs and gut mucosa. Early histological studies revealed lymphoid tissue pathologies (i.e., follicular hyperplasia, follicular lysis, and depletion and fibrosis) that are hallmarks of HIV infection (31, 51). Further work demonstrated a process of progressive deposition of fibrotic collagen, beginning early after HIV infection, driven by TGFβ regulatory CD4 + T cells (34, 52, 53), leading to the loss of stromal cells, like FRCs, and CD4 + T-cell populations (34). Additional significant changes take place in the follicular areas, manifested as enlarged/less-defined follicles and germinal centers, with presumably an important effect on Tfh cell dynamics.

#### Follicular Helper CD4 + T Cells (Tfh)

Follicular helper CD4 + T cells represent a highly differentiated CD4 + T-cell subset with a unique phenotype (4, 40, 41, 54, 55) and molecular signature (4, 39), which provides critical help to follicular B cells during the development of B-cell responses against pathogens and immunogens (44). The chronic phase of HIV/SIV infection is characterized by accumulation of Tfh cells, at least in a group of individuals (4, 56, 57). Furthermore, SIV infection has a significant impact on the gene signature of Tfh cells, characterized by increased expression of IFNγ- and TGFβ-related genes (4). Imaging studies have facilitated the characterization/localization of Tfh cells within the follicular areas during HIV/SIV infection, based on the expression of surface receptors like PD-1 and CXCR5 (4, 8, 13, 58, 59). Furthermore, imaging analysis has revealed that Tfh cells populate different areas of the follicle (marginal zone, surrounding GC, or mainly within the light zone) (Figure 1) (4, 8, 58), presumably exposed to different local signals. Given the dramatic effect of HIV/SIV infection on the LN structure and follicular organization, imaging provides critical information about the distribution of Tfh cells within the follicular areas, as well as their proximity and engagement with B cells. The cellular and molecular mechanisms regulating the dynamics of Tfh cells during HIV/SIV infection are not well understood (4, 60). To this end, tissue imaging can contribute valuable information regarding:

1. The heterogeneity of follicular cells (Tfh subsets like Th1-like Tfh cells (61), dark zone vs. light zone B cells, etc.) and how these populations are impacted during different stages of infection.
2. The possible role of local immune activation/inflammation on T- and B-cell dynamics during HIV/SIV disease progression.
3. The impact of follicular damage/alteration (i.e., loss of FDC) on Tfh and B-cell dynamics.
4. The possible role of locally expressed cytokines (i.e., IL-21, TGFβ, IL-10) or chemokines (i.e., CXCL-13) on Tfh, B-cell dynamics.
5. The distribution of Tfh-infected cells and the dynamics of local HIV/SIV replication.
6. The impact of LN pathologies (i.e., fibrosis, etc.) on LN function (i.e., antigen capture, vaccine responses, etc.).
7. The impact of cART and HIV cure strategies on the FDC reservoir.

#### Follicular CD8 + T Cells

Chronic HIV/SIV infections are characterized by accumulation of CD8 + T cells in the LN and particularly in the follicle, a process referred to as “follicular lysis” (15, 24, 62). Imaging studies have shown that trafficking of virus-specific CD8 + T cells into the follicular area is relatively compromised (63–65). Flow-cytometry-based assays have shown that similar to Tfh cells, follicular CD8 + T cells are characterized by low expression of CCR7 and upregulated CXCR5, and have a unique transcriptional profile (15, 24). What regulates the trafficking of CD8 + T cells, particularly the cytotoxic effector cells, into the LN and distinct microenvironments, like follicles, is not well understood. It was recently shown that expression of viral proteins *per se* may not represent the main force behind this trafficking (15). Imaging analysis can provide critical information regarding the role of local inflammatory cells/signals as mediators of CD8 + T-cell trafficking in the follicular areas during HIV/SIV infection, potentially leading to novel targets for the *in vivo* manipulation of LN CD8 + T-cell dynamics. FRCs provide the cellular network for trafficking of T cells in the T-cell zones (37). Chronic HIV/SIV infection is associated with significant damage to both FRC (34) and follicular structures (31, 33). Whether this tissue damage creates an environment where T-cell trafficking becomes highly stochastic and/or dysfunctional is not known. Thus, imaging studies could be highly informative in addressing these unresolved questions, for example, by assessing the relationship between the magnitude of FDC changes, follicular lysis, and altered chemokine gradients on one hand with follicular CD8 + T-cell enrichment on the other hand.

#### Innate Immunity Cells

Innate immune cells play an important role in HIV/SIV infections and disease at multiple levels, including (i) virus capture and dissemination (66), (ii) expression of pro- and anti-inflammatory mediators (i.e., IFNα/β, TNFα, IL6, IL10, etc.) (67–71), and (iii) expression of pro-inflammatory chemokines (i.e., IP-10, MCP, MIP-1α/β, etc.) (72, 73). Flow-cytometry studies have shown an increased recruitment of hyporesponsive monocyte/macrophages and plasmacytoid dendritic cells early after SIV infection that could affect the ability of IFNα production in the LN (74–76). Complementary to flow-cytometry data, imaging studies have revealed an accumulation of monocytic-lineage cells in areas surrounding the follicle and in close proximity to CD8 + T cells in chronic HIV infection (15) as well as in pathogenic SIV infection of rhesus macaques but not in non-pathogenic SIV infection in nature hosts (i.e., sooty mangabeys) (77). Furthermore, pharmacological manipulation of monocyte activation results in reduced recruitment of activated monocytes to the LN and reduced viral replication (78). More recently, tissue imaging has shown that infected macrophages could contribute to the rapid disease progression in SIV-infected non-human primate (NHP) infants (79). While monocytes/macrophages can clearly become infected with HIV/SIV, the relative contribution of infected monocytes/macrophages as long-lived viral reservoirs *in vivo* is still an open question. Novel, high-resolution imaging approaches allowing for the simultaneous detection of viral RNA and DNA could shed light upon this issue. Furthermore, it is not known if the viral dynamics of infected monocytes/macrophages occurs in a similar fashion in LNs from different anatomical sites, for example, comparing axillary and mesenteric LNs or MALT (80), and warrants further investigation.

Natural killer (NK) cells play an essential role in antiviral immunity, but knowledge of their function in secondary lymphoid organs is incomplete. Contrary to SIV-infected macaques, *in situ* approaches demonstrated that NK cells in secondary lymphoid organs from chronically SIVagm-infected African green monkeys (AGMs) were frequently CXCR5 + and entered and persisted in lymph node follicles where they seem to play a major role in viral reservoir control (81). The relative positioning/compartmentalization of innate cells and associated soluble factors could inform on the role of these cells in the generation and maintenance of effective adaptive immune responses during HIV/SIV.

### The Host: Mucosa

Mucosal barriers are the body’s first defense against external pathogenic threats. Although they represent the boundary between the external environment and the host, mucosal surfaces are often the sites of pathogen transmission (82). In the context of HIV infection, mucosal surfaces represent the major routes of transmission, with the most relevant mucosal tissues being the genital mucosa and gastrointestinal tract (82). Imaging studies utilizing SIV NHP models have been absolutely instrumental in dissecting key aspects of HIV-1 transmission across mucosal surfaces and the early events surrounding mucosal infection, including (i) understanding the unique cellular composition and characteristics of different mucosal tissues and their susceptibility to viral transmission, (ii) defining the early host–viral dynamics within mucosal tissues, including characterizing the principal target cells *in vivo*, and (iii) demonstrating the process and principal pattern of viral dissemination and establishment (83–86).

Disruption of the intestinal barrier and subsequent microbial translocation and inflammation is one of the hallmarks of HIV/SIV pathogenesis and disease progression (87–89). Damaged epithelial integrity (90, 91), as well as the loss of relevant cells from the gut mucosa (92–94), has been associated with HIV/SIV pathogenesis. Imaging studies have been instrumental in investigating the impact of these tissue perturbations in the context of HIV/SIV infections. Besides the documentation of the magnitude of barrier damage, imaging studies have shown: (i) the possible role of gut macrophages with respect to their phagocytic activity (80, 91) or capacity to produce pro-inflammatory cytokines (95) in chronic immune activation and progression to AIDS, (ii) that blocking microbial translocation can reduce viral replication and dissemination in LNs (96), and (iii) that barrier damage and microbial translocation differentiate pathogenic and non-pathogenic SIV infections (91). Furthermore, tissue imaging has been very informative concerning the verification of animal models for SIV pathogenesis––such as the use of pigtail macaques (97) or experimental colitis as an alternative model to investigate the impact of barrier integrity in SIV pathogenesis (16). Similar to LNs, imaging studies will be instrumental in our understanding of the local interplay between the virus and innate/adaptive immunity.

### The Host: Other Tissues

Besides lymphoid organs, other tissues have also been shown to play a role in the pathogenesis of HIV/SIV infections. Imaging studies have been instrumental for our understanding of the CD3 + (98) and CD8 + T cell (99), NK (100) as well as myeloid (Kuppfer) cell (101) dynamics in liver during SIV infection. *In situ* hybridization imaging assays have also shown insufficient viral replication in liver (99, 101). Besides the liver, RNA *in situ* hybridization imaging has been widely used for the detection of cells harboring transcribed virus in several tissues including the following:

(1) adipose tissue and specifically in the stromal vascular fraction (6);
(2) lungs, where macrophages represent a main source of virus production in infant NHP (79, 102) with lung tissue damage associated with infection of interstitial rather than alveolar macrophages (103); and
(3) brain (104) in line with other assays showing that CNS macrophages represents a latent reservoir in cART-treated animals. Confocal imaging of protein markers has revealed the heterogeneity and possible role of monocytes/macrophages, especially recently infiltrating cells, in HIV/SIV encephalitis (105, 106). Increased frequency of perivascular proliferating macrophages (107) could account for the accumulation of macrophages in SIV-infected animals. CNS lesions, found in monkeys receiving cART, were associated with inflammation dominated by lymphocyte and low levels of SIV RNA in the brain (108). Complementary to these imaging studies, use of laser capture microdissection revealed a compartmentalization of viral sequences in brain from animals infected with a neurotropic virus (109). In addition to tissue imaging assays, MRI-based methodologies have been widely used for the study of HIV/SIV neuropahtogenesis (110–113) as well as the *in vivo* viral dynamics in the brain of experimental models (17).

### Host–Virus Interplay

A major obstacle for HIV eradication is the establishment of long-lived viral reservoirs, particularly in “immunologically privileged” areas, like B-cell follicles (114, 115). Therefore, the molecular characterization of cells contributing to these reservoirs, as well as their tissue topology, is of great importance in the development of novel strategies for virus reactivation and elimination. Sensitive PCR-based assays have contributed significantly to our knowledge regarding the dynamics/kinetics of virus replication, the efficacy of cART, and the characterization of cell subsets harboring actively transcribed or latent virus (116–119). Early studies have shown sequestration of viral RNA in follicles using *in situ* hybridization techniques (120). More recently, novel next-generation *in situ* hybridization platforms have been developed with great potential for the comprehensive analysis of viral reservoirs at a tissue level. These platforms allow for the detection of viral RNA (RNAscope) and/or viral DNA (DNAscope) (114, 121, 122). Merging this technology with multispectral confocal microscopy will allow for a comprehensive analysis of (i) the viral reservoir with respect to relevant molecular markers of cells harboring the virus, (ii) the local microenvironment (surrounding immune cells, inflammatory cells, cytokines/chemokines), and (iii) virus dynamics (based on the simultaneous detection of viral RNA, DNA, and viral particles).

In addition to identification of individual cells harboring virus at a tissue level, imaging assays have contributed significantly to our understanding of viral dynamics *in vivo*. Confocal imaging has provided important information regarding viral transmission across and infection in the female reproductive tract (86), as well as revealed that Th17-lineage CD4 + T cells as a preferential target for the virus early after vaginal inoculation (12). Application of technologies like whole-body immune-PET has provided additional insight into the distribution of virus among different organs in chronic SIV infection (123), as well as the impact of antiretroviral or immune-based treatments on viral dynamics (123, 124). Non-invasive whole-body imaging, although of relatively low resolution, provides a “real time” and non-invasive monitoring of viral or relevant immune cell dynamics and could guide the performance of tissue imaging assays for a high-resolution analysis of related cells. Additional information can be obtained from whole-body PET-TDM for drug distribution dynamics (125) allowing the identification of pharmacological sanctuaries, drug interactions and helping the optimization of drug delivery use and drug design.

### Which Imaging Platform?

Today, several imaging technologies and platforms are available for tissue analysis. Several factors should be taken into consideration regarding the choice of the most relevant platform, including the following:

(i) The scientific question under investigation: tissue cell composition and viral reservoirs [light microscopy, confocal microscopy, ion beam imaging (126), Laser Capture Microdissection (127)], subcellular structure and virus–host protein interactions (confocal microscopy, electron microscopy, high-resolution optical imaging technologies), or assessment of cellular and viral dynamics at organ or whole-body level [MRI (17), PET scan (123), confocal endoscopy].
(ii) The requirement for high-resolution, “volumetric” analysis or live imaging (two-photon microscopy) to address the biological process under investigation. The introduction of the two-photon intravital microscopy in the NHP SIV model could revolutionize the field of HIV/SIV pathogenesis and vaccine development by providing real time, *in vivo* measurements of immune cell trafficking, tissue cell dynamics, and interactions between host cells and virus.
(iii) The ability to simultaneously use multiple probes (dimensionality), allowing for the comprehensive analysis of several cells, proteins, and RNA/DNA sequence within the same imaged plane (confocal or imaging mass cytometry). Although high-resolution technologies like electron microscopy-based platforms or super resolution confocal microscopy can provide unprecedented information for the tissue, cell structure, and molecular dynamics, they are lacking their capacity to simultaneously use multiple probes, at least in their current form.
(iv) The potential for “fusion” with other high-throughput platforms. Although current confocal microscopy assays can visualize several probes simultaneously (15), the selection of probes/antibodies is hypothesis-driven. Merging multiplexed confocal microscopy assays with technologies like tissue imaging mass cytometry (128) could provide unique information at multiple levels, including unbiased pathway analysis, discovery of novel therapeutic molecular targets, and pharmacokinetics of antiretroviral regimens.

Modern imaging technologies allow for the acquisition of high-dimensional data. To foster new discoveries derived from such data sets, the development and application of sophisticated algorithms is required. Accurate tissue reconstruction (using advanced 3D tomography algorithms) (129, 130), quantitative analysis of imaging objects using platforms like histocytometry (15, 131), algorithms allowing for the fusion of imaging with other high-throughput platforms (128), as well as modeling tissue cell and virus dynamics based on imaging data could significantly improve our understanding of the highly complex tissue immunobiology, especially during HIV/SIV infection.

Although tissue imaging is a powerful tool, we should keep in mind that there are also limitations that could lead to misinterpretation of tissue immune dynamics. Limited access to tissue material, especially from human subjects, represents a major limitation for tissue analysis. Collecting images from one or two random tissue sections could potentially lead to inaccurate measurements (“sampling error”). Ideally, application of novel, large-volume imaging techniques, like optically cleared tissue imaging (132), could overcome the “sampling error” limitation. However, the need for multiple assays and measurements from usually limited tissue material precludes these types of imaging applications, especially when human tissues are under investigation. Therefore, one should be very cautious with the interpretation of imaging data generated from limited tissue sections. One common practice for the validation of imaging data could be their comparison to data derived from other types of assays, such as flow cytometry.

### Future Directions

Imaging studies have significantly improved our understanding of cellular and molecular mechanisms for HIV/SIV pathogenesis both in humans and NHP SIV models (133). Several imaging studies have validated SIV infection of NHPs as models for HIV pathogenesis, including the illustration of early events resulting in HIV/SIV transmission (86, 134, 135), the documentation and role of gut mucosal barrier damage in HIV/SIV pathogenesis (89, 91), as well as the impact of infection in secondary lymphoid tissues (31). Particularly for lymph node dynamics, a similar profile for follicular CD4 + (4, 56, 57) and CD8 + T cells (15, 24) has been shown in infected humans and NHPs, while *in situ* hybridization assays have established the importance of these sites for virus persistence (64, 122). Given the difficulty in obtaining human tissues from different anatomical sites, the performance of imaging studies in SIV models will continue to provide unpresented information regarding the anatomical compartmentalization of these immune dynamics.

We are witnessing a boom of imaging technologies that expand our capacity for comprehensive spatial analysis of tissue cells and molecules with high definition. Given the complexity of tissue immunobiology, the performance of different imaging-based assays, as well as their merging with other high-throughput assays, is of great importance for the generation of high-dimensional data. Besides the characterization of virus and cells at the tissue level, imaging technologies could prove useful in the analysis of other biological parameters, such as metabolic status, monitoring of therapeutics (pharmacokinetic studies), or novel immunotherapies (i.e., administration of multi-specific antibodies). Although HIV/SIV infections lead to major changes in tissue architecture, imaging the immune system in infected humans and NHPs can potentially provide insight into the overall anatomy and organization of the immune system in disease and contribute to the generation of a human cellular atlas.

## Author Contributions

All authors have contributed equally to this work.

## Conflict of Interest Statement

The authors declare that the research was conducted in the absence of any commercial or financial relationships that could be construed as a potential conflict of interest.
